# Azelaic acid stimulates catalase activation and promotes hair growth through upregulation of* Gli1* and *Gli2* mRNA and Shh protein

**Published:** 2020

**Authors:** Elham Amirfakhryan, Behzad Davarnia, Farhad Jeddi, Nowruz Najafzadeh

**Affiliations:** 1 *Research Laboratory for Embryology and Stem Cells, Department of Anatomical Sciences and Pathology, School of Medicine, Ardabil University of Medical Sciences, Ardabil, Iran *; 2 *Department of Biochemistry, School of Medicine, Ardabil University of Medical University, Ardabil, Iran*

**Keywords:** Hair follicle, Minoxidil, Azelaic acid, Anagen, PCR, Immunocytochemistry

## Abstract

**Objective:**

Although azelaic acid is effective for treatment of acne and rosacea, the biological activity of azelaic acid and the effect of its combination therapy with minoxidil were not elucidated with regard to hair growth.

**Materials and Methods:**

In this study, mouse vibrissae follicles were dissected on day 10 after depilation. Then, the bulb and bulge cells of the hair follicle were treated with minoxidil and azelaic acid for 10 days to evaluate Sonic hedgehog (Shh) protein expression. Moreover, bulge and bulb cells of the hair follicles were cultivated and the expression of *Gli1*,* Gli2, *and* Axin2 *mRNA levels was evaluated using real-time polymerase chain reaction (PCR) analysis. We further investigated the protective effects of azelaic acid against ultraviolet B (UVB) irradiation in cultured bulb and bulge cells by determining catalase activity. An irradiation dose of 20 mJ/cm^2^ UVB for 4 sec was chosen.

**Results:**

The results showed that catalase activity significantly (p<0.05) increased in the bulge cells after exposure to 2.5 mM and 25 mM azelaic acid. Meanwhile, treatment of the bulb cells with azelaic acid (2.5 and 25 mM) did not cause significant changes in catalase activity. We also found that azelaic acid (25 mM) alone upregulated *Gli1 *and *Gli2* expression in the bulge cells and 100 µ minoxidil caused *Gli1* and *Axin2* overexpression in the bulb region of the hair follicle. Moreover, minoxidil (100 µM) alone and in combination with azelaic acid (25 mM) led to Shh protein overexpression in the hair follicles *in vitro* and in organ culture.

**Conclusion:**

Our results indicated a potential role for azelaic acid in the protection of bulge cells from UVB damage and its combination with minoxidil may activate hair growth through overexpression of Shh protein.

## Introduction

Hair follicle undergoes cyclic morphogenetic changes of anagen, catagen, and telogen, throughout life (Krause and Foitzik, 2006 ▶). These cyclic changes involve interactions between epithelial and dermal cells of the hair follicle (Sennett and Rendl, 2012 ▶). There are multipotent stem cells with self-renewal capability in the bulge region of the hair follicles (Najafzadeh et al., 2015 ▶). The bulge stem cells can migrate to the lower part of the hair follicle within the outer root sheath and reconstitute the matrix cells at the end of telogen (Myung and Ito, 2012 ▶). The bulge stem cells are slow cycling cells (Cotsarelis et al., 1990 ▶) and regenerate lower half of the hair follicle during the anagen phase (Morris et al., 2004 ▶). 

The hair follicle stem cells (HFSCs) residing in the bulge region are maintained by activation of Wnt and sonic hedgehog (Shh) signaling pathways. Shh directs patterning and cellular differentiation during embryonic development of the skin and hair follicle. Moreover, Shh is involved in controlling anagen stage of hair follicle during postnatal life (Chiang et al., 1999 ▶). In vertebrates, three Gli proteins, Gli1, Gli2, and Gli3 are involved in Shh signaling. Gli1 is a transcriptional activator, whereas Gli2 and Gli3 act as either activator or repressor. Gli2 protein plays a critical role during the development of the hair follicles. It induces cell proliferation in the hair follicles by activating *cyclin D1* and *cyclin D2* (Mill et al., 2003 ▶). The dermal papilla and outer root sheath cells of the hair follicle express* Gli1* and *Gli2* during anagen (Paladini et al., 2005 ▶). The Wnt target gene *Axin2* expression persists in the bulge stem cells during the initiation of hair growth phase (Lim et al., 2016 ▶).

Androgenetic alopecia** (**AGA) affects about 30-50% of men and 13-37% of women by the age of 50 years old (Hamilton, 1951 ▶; Venning and Dawber, 1988 ▶). AGA is related to genetic and androgens including testosterone and dihydrotestosterone (DHT). DHT is a more potent androgen that can activate genes responsible for scalp hair follicle miniaturization (Inui and Itami, 2011 ▶). In AGA, hair growth is ceased while telogen duration prolongs and leads to the conversion of terminal hairs into vellus hairs (Otberg et al., 2007 ▶). Based on such knowledge, minoxidil induces the initiation of the anagen phase and prolongs its duration (Messenger and Rundegren, 2004 ▶). Minoxidil stimulates peripheral vasodilation around the hair follicle and promotes dermal papilla cells survival and proliferation (Han et al., 2004 ▶). It can also induce prostaglandin-endoperoxide synthase-1 and upregulate vascular endothelial growth factor (Lachgar et al., 1998 ▶).

In recent years, due to negative psychosocial impact of alopecia, patients turn to alternative medicine that has not yet been clarified for their effectiveness. Recently, the beneficial effects of herbal extracts such as pumpkin seed oil (Hajhashemi et al., 2019 ▶), *Citrullus colocynthis*, and *Rosmarinus officinalis *were evaluated (Hosking et al., 2019 ▶). Azelaic acid was previously used in the treatment of rosacea, acne, and melasma (Frampton and Wagstaff, 2004 ▶). In human keratinocytes, azelaic acid induces *PPARγ* transcriptional activity (Mastrofrancesco et al., 2010 ▶) and suppresses *IL-1β*, *IL-6*, and *TNF* mRNA expression. In earlier studies, it was shown that azelaic acid reduces inflammation and may act as a potent 5α-reductase inhibitor (Stamatiadis et al., 1988 ▶) which may reduce hair loss. Azelaic acid has not been extensively studied as a treatment for hair loss. In many products, azelaic acid has been combined with minoxidil to treat AGA (Hordinsky and Donati, 2014 ▶). Some dermatologists prescribe commercial solutions supplemented with azelaic acid for AGA (Sasmaz and Arican, 2005 ▶). A great part of azelaic acid effect against hair loss may be related to its anti-inflammatory properties; however, there is little evidence to prove the efficacy of azelaic acid on hair growth. Targeting and inhibiting DHT and inflammation would be a rational approach for developing novel therapeutics for treatment of hair loss. In this study, we hypothesized that induction of the overexpression of *Gli1*, *Gli2*, and *Axin2* in hair follicle by azelaic acid may initiate hair regrowth. Overall, there is a need for additional studies to evaluate possible mechanism underlying azelaic acid effect by evaluating the expression of Shh protein and the anagen promoting genes such as *Gli1*, *Gli2*, and *Axin2 *in the hair follicles.

## Materials and Methods

**Drugs and reagent**

Minoxidil and azelaic acid were purchased from Sigma-Aldrich (St Louis, MO, U.S.A). Minoxidil was dissolved in HCl and azelaic acid was dissolved in culture medium.

**Animals**

Male BALB/c mice (6-8 weeks old) were obtained from Razi Vaccine and Serum Research Institute (Karaj, Iran). The animals were kept under a 12:12 hr light/dark cycles and they had free access to food and water. The experimental procedures were performed in accordance with the guidelines of the Animal Care and Use Committee of Ardabil University of Medical sciences (IR.ARUMS.REC.1394.50).

**Isolation of bulb and bulge regions**

All mice upper lip hairs were depilated to synchronize the hair follicles in anagen. Ten days later, the whisker pads were collected. Briefly, after anesthesia induction using dimethyl ether, anagen stage-follicles were dissected from the whisker pads under a stereomicroscope (Olympus, Tokyo, Japan). The vibrissa hair follicles lifted out and treated with minoxidil (100 μM), azelaic acid (25 mM) alone and azelaic (25 mM)/minoxidil (100 µM). Then, the bulge regions were isolated from the hair follicles. Bulb region was also isolated by cutting a piece of around 2–2.5 mm in length from the bottom of the hair follicle (Dastan et al., 2016 ▶).

**UVB irradiation**

Bulb and bulge cells of the hair follicle were cultured in Dulbecco’s Modified Eagle’s medium: nutrient mixture Ham’s F12 medium (DMEM: F12). Prior to the irradiation, the medium was replaced by *phosphate buffered saline (*PBS). An irradiation dose of 20 mJ/cm^2^ UVB was chosen based on previous results (Mastrofrancesco et al., 2010 ▶). The average irradiation time was 4 sec. After irradiation, the cells were immediately returned to the culture medium with or without addition of azelaic acid (2.5 and 25 mM for 7 days). Sham cells were irradiated, but control cells were incubated in DMEM/ F12 medium without UVB exposure.

**Determination of catalase activity**

The bulb and bulge regions were isolated and the cells were cultivated in DMEM: F12 supplemented with 100 U/ml penicillin and 100 µg/ml streptomycin. The groups were: (i) control (untreated bulb and bulge cells); (ii) Sham (untreated bulb and bulge cells+UV irradiation); (iii) UV+2.5 mM azelaic acid; (iv) UV+25 mM azelaic acid; (v) 2.5 mM azelaic acid+UV; (vi) 25 mM azelaic acid+UV. 

Catalase activity was measured according to Aebi method (Aebi, 1984). Briefly, the bulb and bulge cells were washed and suspended in 0.1 M Tris-HCl (pH 7.5) for 20 min on ice. Then, the cells were sonicated for two 30-sec bursts. After 10 min of centrifugation at 14000 relative *centrifugal* force (RCF), aliquots of the obtained supernatant were subjected to three freeze-thaw cycles at -80°C. Next, 50 μl supernatant was diluted 500 times with PBS and 2 μl of that was added to 1 ml hydrogen peroxide (30 mM). The absorption was monitored by a spectrophotometer (Shimadzu, UV-1800) at λ=240 nm every 15 sec for 2 min. Assays were performed in triplicate.

**Immunoassay**

Immunohistochemistry was performed as described previously (Najafzadeh et al., 2013 ▶). Briefly, ten-micron sections were prepared, mounted on slides and deparaffinized. After rehydration, the sections were incubated in blocking solution (PBS with 5% normal goat serum, 0.3% Triton X-100 and 1% bovine serum albumin) at room temperature for 60 min. The cells were incubated with primary antibody against Shh (sc-9024; Santa Cruz, 1:100). Then, the cells were stained with the secondary antibody (donkey anti-rabbit Alexa Fluor 488 (Invitrogen, A21206, 1:200) (Najafzadeh et al., 2015 ▶). For immunocytochemistry, after fixation using paraformaldehyde, the cells were blocked and stained with primary antibody (anti-Shh (1:100)) and secondary antibody (Alexa Fluor 488 (1:200)). The cells were visualized by a fluorescence microscope (IX71, OLYMPUS).

**Quantitative real-time PCR (qRT-PCR) analysis**

The anagen hair follicles were treated with minoxidil, azelaic acid and the combination of them for 10 days. In all experiments, the tissue culture medium containing minoxidil or azelaic acid, was changed every two days. Then, the follicular bulge and bulb regions were isolated for real-time PCR analysis. Total mRNAs was extracted from the hair follicles using TRIzol reagent (Invitrogen, Carlsbad, CA). The concentration of total mRNA was measured using a NanoDrop Spectrophotometer (Thermo Scientific, USA). cDNA was synthesized from total RNA using a cDNA Synthesis Kit (Fermentas, Thermo Fisher Scientific, USA). Reactions were performed in a final volume of 20 μl (master mix 10 μl, cDNA 4 μl, primer 2 μl, and H_2_O 4 μl). The primer sequences and products lengths are shown in Table 1. Quantitative PCR was carried out using Power SYBR Green PCR Master Mix (EURx, Ltd, Gdañsk, Poland) and real-time PCR System (Applied Biosystems 7500, Foster City, CA, USA). The cycling initiated with denaturation at 95°C for 10 min, followed by 50 cycles of denaturation at 95°C for 10 sec, annealing at 60°C for 20 sec and extension at 72°C for 10 sec. Β2-microglobulin (*B2M*) gene was applied as the housekeeping gene for normalization of the real-time data. The 2^−ΔΔCt^ formula was used to calculate relative changes in gene expression (Dastan et al., 2016 ▶; Mohammadi Jobani et al., 2018 ▶).

ΔΔCt = (ΔCt_(sample)_ – ΔCt_ (control)_)

**Statistical analysis**

Statistical analysis was done by one-way analysis of variance (ANOVA). p values<0.05 were regarded as statistically significant. Graphs were plotted using Sigma Plot software (version 12.0, Systat Software Inc., SanJose, CA).

## Results

**Azelaic acid induced catalase activity in bulge cells**

**Figure 1:**
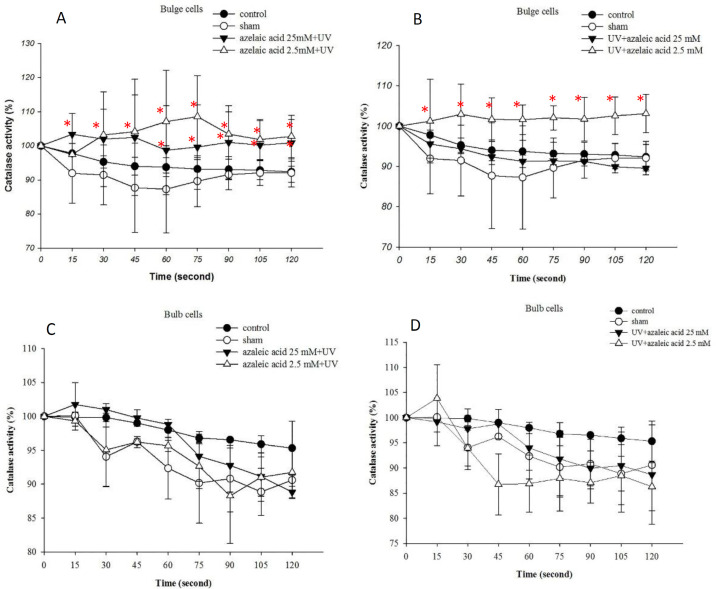
Induction of catalase activity after azelaic acid (2.5 and 25 mM) treatment in the bulge (A and B) and bulb cells (C and D). Azelaic acid was added 30 min before UVB (20 mJ/cm^2^) (A and C) or promptly after UVB radiation (B and D). ^*^p<0.05 when compared to control and sham

To evaluate the short-term sensitivity of hair follicle to UVB exposure, we irradiated the bulge and bulb regions of the hair follicle with a single dose of UVB. When azelaic acid (2.5 mM) was added into culture medium before UVB irradiation or when added promptly after irradiation, it significantly increased catalase activity in the bulge cells (Figure 1A). The increase in the catalase activity was also promoted by 25 mM azelaic acid in the bulge cells before UVB irradiation (Figure 1B). Treatment of the bulb cells with azelaic acid (2.5 and 25 mM) did not cause significant changes in the catalase activity (Figures 1C and D).

**Table 1:** Sequences of the primers used to identify different genes in real-time PCR assays

**Name**	**Forward Primer (5’-3’)**	**Reverse Primer (5’-3’)**	**Product length**
***Gli1***	ACTAGGGGCTACAGGAGGA	ACCTGGACCCCTAGCTTCAT	149 bp
***Gli2***	ACCATGCCTACCCAACTCAG	CCTCAGCCTCAGTCTTGACC	145 bp
***Axin2***	AACCTATGCCCGTTTCCTCT	CTGGTCACCCAACAAGGAGT	128 bp
***B2M***	CTGCTACGTAACACAGTTCCACCC	CATGATGCTTGATCACATGTCTCG	241 bp

**Minoxidil (100 µM) and its combination with** **azelaic acid (25 mM) increased the expression of Shh protein**

**Figure 2:**
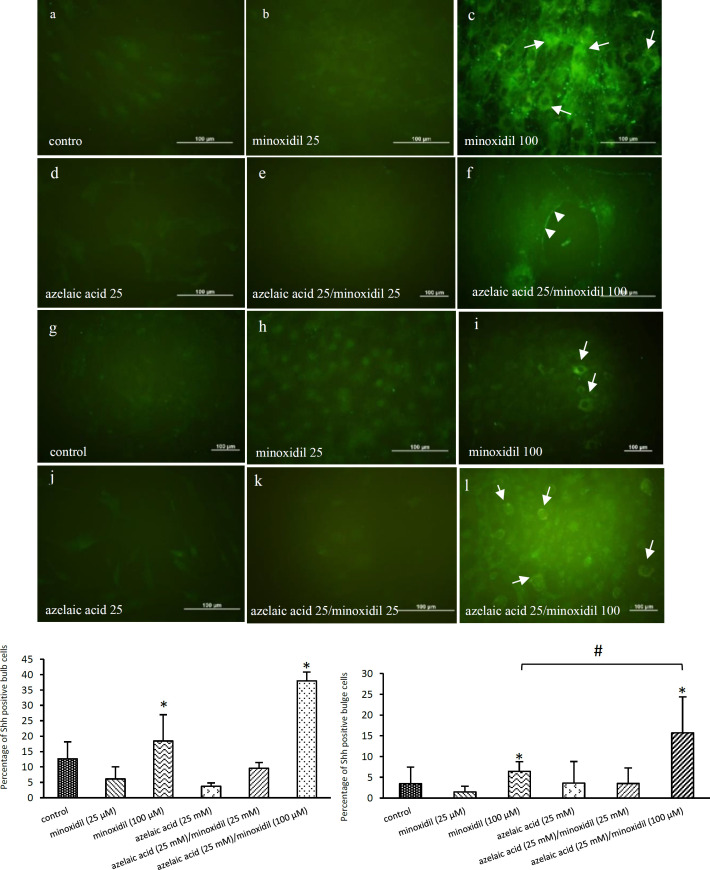
Effects of minoxidil, azelaic acid, and the combination treatments on Shh expression in the bulb (a-f) and bulge (g-l) cells of the hair follicle. Bulb and bulge cells were cultured in medium without drugs (control, a and g), or with azelaic acid (25 mM), minoxidil (25 and 100 μM), azelaic acid (25 mM)/minoxidil (25 µM), and azelaic acid (25 mM)/minoxidil (100 µM). Shh expression was apparently seen in both minoxidil (100 µM) treated bulb (c and m) and bulge (i and n) cells. Indeed, azelaic acid (25 mM) combination with minoxidil (100 µM) significantly induced more Shh expression in the bulb (f and m) and bulge cells (l and n) (^∗^p<0.05). Azelaic acid (25 mM), minoxidil (25 µM), and azelaic acid (25 mM)/ minoxidil (25 μM) had no effect on Shh expression. *p<0.05 when compared to the control. #p<0.05 when compared to minoxidil (100 µM).

**Figure 3:**
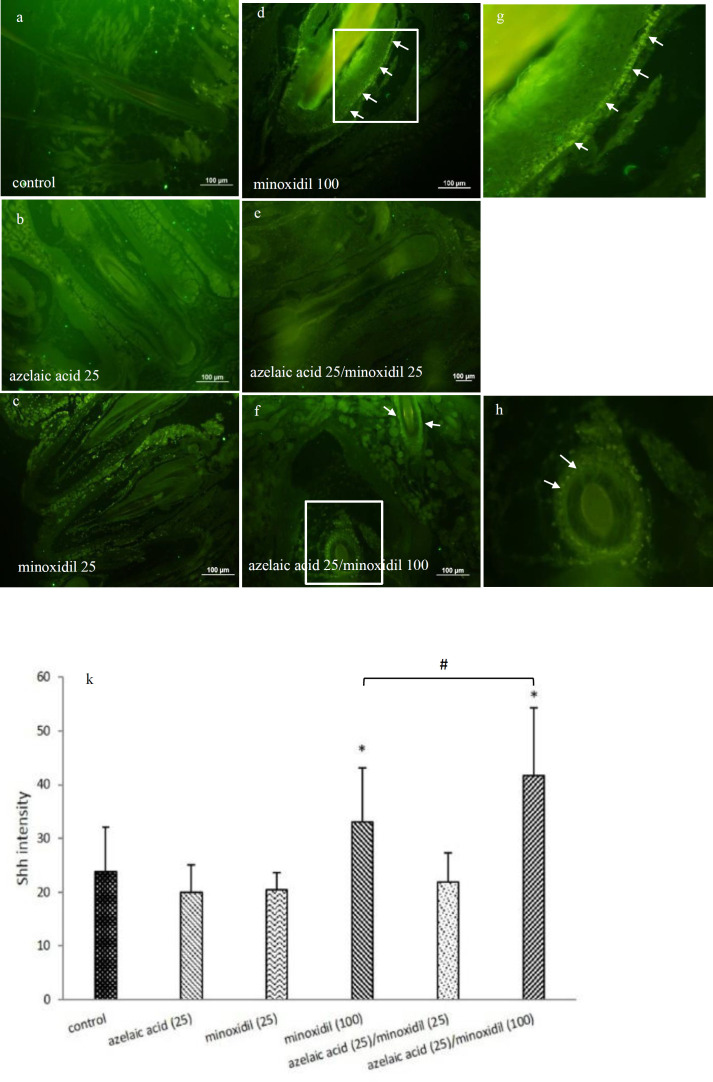
Effects of azelaic acid, minoxidil and the combination treatments on Shh expression in the hair follicles. Azelaic acid (25 mM) (b), minoxidil (25 µM) (c), and azelaic acid (25 mM)/minoxidil (25 µM) (e) had no significant effect on Shh expression, but minoxidil (100 µM) (d and g) and azelaic acid (25 mM)/minoxidil (100 µM) (f, h, and k) treatments resulted in a significant increase in Shh protein expression in the hair follicles (*p<0.05). The arrows show Shh immunostaining in the outer root sheath of the hair follicles. *p<0.05 when compared to the control. #p<0.05 when compared to minoxidil (100 µM)

To elucidate whether the treated hair follicles undergo anagen phase, we tested the expression of Shh protein using immunoassay. Our results showed that Shh protein expression was increased in 100 µM minoxidil-treated cells compared to the control cells (untreated) on day 10 post-treatment. In addition, as shown in Figure 2, azelaic acid (25 mM)/minoxidil (100 µM) combination treatment significantly increased Shh expression in the bulge and bulb cells when compared to control and minoxidil (100 µM). Minoxidil (100 µM) and azelaic acid (25 mM)/minoxidil (100 µM) treatments also induced Shh protein expression in the outer root sheath of the hair follicles, while other treated groups lacked expression or had low levels of Shh protein expression (Figure 3).

**Table 2:** mRNA expression levels of *Gli1, Gli2, *and *Axin2 *in the bulb and bulge regions of the hair follicles were analyzed by real time-PCR. The relative mRNA expression of different genes is presented as fold changes

		**Bulge region**			**Bulb region**	
**Gene**	azelaic (25 mM)	minoxidil (100 µM)	azelaic (25 mM)/minoxidil (100 µM)	azelaic (25 mM)	minoxidil (100 µM)	azelaic (25 mm)/ minoxidil (100 µM)
***Gli1***	168.19±35.99*	0.08±0.03	0.66±0.10	0.42±0.08	5.19±1.31*	0.056±0.012
***Gli2***	9.72±1.58*	1.13±0.59	0.159±0.05	0.0034±0.0016	0.117±0.045	0.041±0.027
***Axin2***	0.86±0.23	0.19±0.09	0.157±0.11	0.023±0.0077	5.37±0.32*	0.021±0.005

*Gli1* and *Gli2* mRNA levels are statistically significant in azelaic treated bulge cells. *Gli1* and *Axin2* upregulated in minoxidil treated bulb cells (*P< 0.05 compared to control).

**Effects of minoxidil, azelaic acid and the combination treatments on** ***Gli1*** **,** ***Gli2*** **, and** ***Axin2*** **expression**

In our study, we explored the effect of azelaic acid (25 mM), minoxidil (100 µM) and azelaic acid (25 mM)/minoxidil (100 µM) on *Gli1, Gli2,* and *Axin2 *mRNA expression in the bulb and bulge regions of the hair follicles. The fold changes of *Gli1 *and* Gli2 *mRNA levels were 194.81 and 10.82 in the azelaic acid-treated bulge cells, respectively. In addition, 100 µM minoxidil-treated bulb cells showed a significant increase in *Gli1* (6.026-fold change) and *Axin2 *(5.15-fold change) mRNA level (Table 2). In contrast, minoxidil plus azelaic acid did not elevate the level of the gene expression compared to minoxidil alone, in bulge and bulb cells of the hair follicles.

## Discussion

In our results, the effectiveness of azelaic acid alone in inducing hair growth, was shown with respect to up-regulation of *Gli1 *and* Gli2 *genes and an increase in catalase activity in the bulge cells. Indeed, its combination with minoxidil also elevated Shh protein expression in the hair follicles and combination therapy had a higher effect over minoxidil alone in terms of increased Shh expression level. Similar to our results, previous studies showed that Shh/Gli1/Gli2 activation may directly promote telogen to anagen transition in the hair follicle (St-Jacques et al., 1998 ▶) and azelaic acid may exert various PPAR*γ*-independent effects in the hair follicle. Moreover, direct upregulation of *Gli2 *shown here and by other studies (Mill et al., 2003 ▶; Oro and Higgins, 2003 ▶) as *Gli2 *is a transcriptional partner for *β*-catenin, that acts in synergy to induce anagen promotion and hair growth morphogenesis (Reddy et al., 2001 ▶). Previously, Pantazi et al. showed that Gli2 promotes the survival of the keratinocytes by inhibiting apoptosis and deregulating cell cycle proteins (Pantazi et al., 2015 ▶).

Several signals were previously shown to participate in the hair follicle cyclic changes*. β*-catenin, Shh, noggin, and STAT3 are key factors that trigger the anagen phase (Botchkarev and Kishimoto, 2003 ▶). Shh/Gli regulate hair follicle cyclic growth by inducing telogen to anagen transition (Choi, 2018 ▶). Importantly, Gli1 protein is expressed in LGR5- expressing hair follicle stem cells (Brownell et al., 2011 ▶). Similar to our results, some studies attempted to use azelaic acid to treat androgenetic alopecia and telogen effluvium. Comparison of azelaic acid and anthralin for treatment of patchy alopecia areata showed that azelaic acid and anthralin gave similar results to hair regrowth (Sasmaz and Arican, 2005 ▶). Consistent with our study, Pazoki-Toroudi et al showed that topical use of azelaic acid combination with 12.5% minoxidil was more effective for hair regrowth and produced a significant decrease in shedding (Pazoki-Toroudi H et al., 2012 ▶). In another study, Gugle et al. demonstrated that minoxidil alone and a combination of minoxidil, azelaic acid, and tretinoin were equally effective in treatment of androgenetic alopecia and the combination therapy had no added advantage over minoxidil alone (Gugle et al., 2015 ▶).

Furthermore, we showed that exposure of azelaic acid to bulge cells protected them from UVB damage and caused a significant increase in the catalase activity in the bulge area. Hair loss, like acne, is strongly linked to inflammatory factors and DHT (Knussmann et al., 1992 ▶). In scarring alopecia, hair follicle stem cells (bulge cells) degenerate resulting in permanent hair loss (Harries and Paus, 2009 ▶). Some PPARγ ligands such as azelaic acid, have anti-inflammatory activity for the treatment of cicatricial (scarring) alopecia (Mirmirani and Karnik, 2009 ▶). It inhibits neutrophil-mediated reactive oxygen species (ROS) production (Akamatsu et al., 1991 ▶) and modulates the inflammatory process.

Finally, we found that *Axin2 *mRNA level was significantly up-regulated in the bulb cells after 100 µM minoxidil treatment. Axin2 and PPARγ participate in a negative feedback loop to degenerate and inhibit* β*-catenin (Lecarpentier and Vallée, 2016 ▶; Stamos and Weis, 2013 ▶). The degeneration of *β*-catenin in the cytoplasm protects the activation of target genes such as *cyclin D* and *c-myc *(Tetsu and McCormick, 1999 ▶). Most signaling pathways contain negative feedback loops, which silence the signaling after the initial stimuli (Jho et al., 2002 ▶).

Our previous work revealed that minoxidil promotes anagen phases through up-regulation of *β-catenin *and *Shh *(Dastan et al., 2016 ▶). Transient activation of the Wnt signaling pathway is needed for the initiation of anagen and activation of bulge stem cells in the hair follicles (Lien et al., 2014 ▶). Probably, *Axin2* gene inhibits Wnt signaling in the bulb cells in a negative feedback mechanism after anagen initiation (Lim et al., 2016 ▶). Similar to our study, Kwack et al. demonstrated that minoxidil induces accumulation of *β-*catenin and up-regulates the expression of Axin2, Lef-1, and EP2 (Kwack et al., 2011 ▶). In addition to activation of Wnt signaling, Shh is required for anagen progression of the hair follicle (Wang et al., 2000 ▶) and it may influence the bulb cells of hair follicles to express *Gli1* and *Axin2*.

Shh signaling regulates dermal papilla fibroblasts and matrix keratinocytes interaction during embryonic follicle morphogenesis and postnatal hair follicles cycling (Callahan and Oro, 2001 ▶). Shh is expressed in the keratinocytes of hair matrix and it acts as a mitogen to induce anagen (St-Jacques et al., 1998 ▶).

In conclusion, we showed that azelaic acid is a regulator of *Gli1* and *Gli2 *genes and it can protect bulge cells from UVB damage. Our results suggest that azelaic acid combination with minoxidil promotes hair growth via induction of Shh protein expression in the hair follicles. Altogether, this evidence provides valuable insights into the role of azelaic acid in the hair follicles growth. 
